# Smoking and secondhand smoke among patients with systemic lupus erythematosus and controls: associations with disease and disease damage

**DOI:** 10.1186/ar4656

**Published:** 2014-09-18

**Authors:** Samantha J Minkin, Stephanie N Slan, Gary S Gilkeson, Diane L Kamen

**Affiliations:** 1 Medical University of South Carolina, Charleston, SC, USA

## Background

Previous reports suggest smoking may be a risk factor for developing systemic lupus erythematosus (SLE). This study explores the impact of tobacco smoke on SLE patients compared with controls and on disease characteristics among patients.

## Methods

Data from a cohort of SLE patients and controls were utilized. Medical history, smoking and secondhand smoke exposure history, SLE Disease Activity Index (SLEDAI) and SLICC Damage Index (SDI) scores were collected at an in-person enrollment visit and confirmed by chart review. Statistical analysis used chi-square testing and multivariate logistic regression.

## Results

There were 545 SLE patients and 386 controls with data available for analysis (Table 1). At enrollment, the mean age was 37.6 ± 14.7 years for patients and 42.0 ± 15.4 years for controls. Differences between current and never smokers (*P *= 0.51) and ever and never smokers (*P *= 0.70) were not significantly different between patients and controls. Compared with unrelated controls, African American patients were significantly more likely to be exposed in the home to secondhand smoke before the age of 18 (OR 1.81, 95% CI 1.13 to 2.89). Damage by SDI (SDI > 0) was significantly associated with ever smoking (OR 3.08, 95% CI 1.4 to 6.6), current smoking (OR 3.17, 95% CI 1.1 to 9.1), and secondhand smoke exposure in childhood (OR 1.91, 95% CI 1.0 to 3.6). No significant relationship was found between smoking status and active disease at enrollment (SLEDAI ≥ 6) or dsDNA autoantibodies. Discoid rash was significantly associated with ever smoking (OR 2.74, 95% CI 1.5 to 5.1) and current smoking (OR 4.85, 95% CI 2.2 to 10.5).

**Table 1:** 

	**Never smokers, *n ***(%)	**Ever smokers, *n ***(%)	**Current smokers, *n ***(%)	**Secondhand smoke < 18 years old, *n ***(%)	**Secondhand smoke ever, *n ***(%)
All patients (*n *= 545)	407 (74.7%)	138 (25.3%)	72 (15.1%)	132 of 372 (35.5%)	158 of 376 (42.0%)
African American patients (*n *= 416)	328 (78.9%)	88 (21.2%)	49 (11.8%)	111 of 313 (35.5%)	127 of 315 (40.3%)
Caucasian patients (*n *= 109)	63 (57.8%)	46 (42.2 %)	21 (19.3%)	19 of 47 (40.4%)	26 of 47 (55.3%)
Other patients (*n *= 20)	16 (80%)	4 (20.0%)	2 (10%)	2 of 12 (16.7%)	5 of 14 (35.7%)
All controls (African American) (*n *= 386)	284 (73.6%)	102 (26.4%)	57 (14.8%)	92 of 354 (25.6%)	120 of 357 (33.6%)
Related controls (*n *= 222)	155 (69.8%)	67 (30.2%)	36 (16.2%)	51 of 205 (24.9%)	67 of 207 (32.4%)
Unrelated controls (*n *= 164)	129 (78.7%)	35 (21.3%)	21 (12.8%)	41 of 149 (27.5%)	53 of 150 (35.3%)

## Conclusions

Our study suggests that secondhand smoke during childhood may be a risk factor for SLE. Secondhand smoke during childhood, current smoking and past smoking contribute significantly to disease damage among patients with SLE.

